# Unlocking the impact of citric acid and sugar on the nutritional, physicochemical, microbial and sensorial attributes of mango fruit leathers

**DOI:** 10.1016/j.fochx.2025.103478

**Published:** 2025-12-31

**Authors:** Muhammad Muzamil, Raheel Suleman, Muhammad Waseem, Tariq Ismail, Muhammad Shoaib, Muhammad Qamar, Muhammad Aftab Zahoor, Tawfiq Alsulami, Robert Mugabi

**Affiliations:** a Department of Food Science and Technology, Faculty of Food Science and Nutrition, Bahauddin Zakariya University, Multan 60800, Pakistan; b College of Ocean Food and Biological Engineering, Jimei University, Xiamen 361021, China; c Department of Food Science and Technology, Faculty of Agriculture and Environment, The Islamia University of Bahawalpur, 63100, Pakistan; d College of Biosystems Engineering and Food Science, Zhejiang University, 866 Yuhangtang Road, Hangzhou 310058, PR China; e Department of Food Science & Nutrition, College of Food and Agricultural Sciences, King Saud University, Riyadh 11451, Saudi Arabia; f Department of Food Technology and Nutrition, Makerere University, Kampala, Uganda

**Keywords:** *Mangifera indica*, Fruits, Leather, Value addition, Confectionery, Sensory

## Abstract

This study investigated the effects of citric acid and sugar on the quality of mango fruit leather during 0–90 days of storage. The nutritional and textural properties of mango fruit leathers showed a significant increase (*p < 0.05*) in ash, K, Mg, vitamin C i.e., 1.9–2, 270–275, 21–23, and 22–24 mg/100 g, respectively, while a decrease in moisture and hardness of samples i.e., 17–15 % and 8.6–5.6 N in MFL_0_ (i.e., zero citric acid and sugar) to MFL_CS_ (i.e., 0.2 % citric acid+10 % sugar), respectively. DPPH activity, total phenolic content (TPC) and total flavonoids content (TFC) significantly (*p < 0.05*) increased from 48.2 to 61.5 %, 170–400 mg GAE/g and 126–163 mg CE/g in MFL_0_-MFL_CS_, respectively. Likewise, scanning electron microscopic (SEM) analysis reported significant differences in surface morphology in each treatment. *L*^*⁎*^, *a*^*⁎*^ and *b*^*⁎*^ values were notably decreased from 52.7 to 46, 17–12 and 29–16 in MFL_0_-MFL_CS_, respectively at 0–90 of storage. On storage, pH deceased from 4.1 to 3.7 and titratable acidity increased from 0.5 to 0.6 in MFL_0_-MFL_CS_, respectively. Total plate counts (TPC), mold and yeast count elucidated measurable decrease form 3.4–3 and 2–1.7 at 90th day of storage, respectively. Sensory panelists gave the highest sensory scores to MFL_CS_ (i.e., 0.2 % citric acid+10 % sugar) for its superior sensory attributes and overall acceptability. Conclusively, MFL_CS_ showed the best results for all parameters; however, further studies are recommended to develop and evaluate fruit leathers for quality using different fruits at multiple concentrations of citric and sugar.

## Highlights

• Mango leather nutritional profile improved with addition of citric acid and sugar.
• Phenolic and flavonoids content increased with citric acid and sugar addition.
• Total plate, mold and yeast counts decreased with addition of citric acid and sugar.
• Best organoleptic properties observed with 0.2 % citric acid+10 % sugar over 90 days.
• Synergistic effect of citric acid and sugar addition enhanced storage life.

## 1 Introduction

Mango (*Mangifera indica L.*) is an important fruit of subtropical and tropical regions and belongs to the family *Anacardiacea*. It is widely consumed due to its appealing color, pleasant taste, sweet aroma, high palatability and nutritional value (Kabir et al., 2024). Mango production is increasing steadily, making it predominant fruit of 21st century and worldwide production of mangoes extended to 51 million tons in 2019 in India, Pakistan, China, Thailand and Indonesia. India is considered the top producer of mangoes with its production reaching to 22 million tons per year (Kaur et al., 2023). Mangoes are also essential carrier of macronutrients i.e., essential proteins, low calorie carbohydrate, dietary fibers, essential lipids, and micronutrients i.e., vitamin C, vitamin A, potassium, phosphorus, calcium, magnesium, sodium, iron, manganese and copper (Lebaka et al., 2021).

Botanical composition of mangoes reveal an array of bioactive compounds such as phenolics, carotenoids, polysaccharides, alkaloids and sterols (Boudon et al., 2020). It contains phenolic acids (i.e., vanillic acid, gallic acid, proto-catechuic acid, syringic acid, *p*-coumaric acid, caffeic acid, chlorogenic and ferulic acid), carotenoids (i.e., zeaxanthin, cryptoxanthin, violaxanthin and lutein) and flavonoids i.e., mangiferin, quercetin, kaempferol, tannic acid, anthocyanins, ascatechins and rhamnetin (Lebaka et al., 2021; Maldonado-Celis et al., 2019), playing their significant role as antimicrobials, antioxidants, anti-inflammatory, antiallergic, antitumor, antiaging, cardio-protective, neuroprotective, hepatoprotective and vision-protective agents (Kumar et al., 2021). Mangoes are climacteric fruits and have short shelf life of 3 to 4 days depends upon its variety and storage conditions (Ullah et al., 2025).

Lack of experience, improper handling and inappropriate storage results in global food losses of mangoes reach up to 50 % annually (Bisht & Singh, 2024), which therefore Processing of fruits into value-added products can be considered as a key step to enhance its economic value (Sukasih & Widayanti, 2022). Therefore, scientists are in search of natural potential ingredients for value-addition of fruits and vegetables to add value in shelf life, economic significance and novel food products. In this context, previous studies have reported the use of mangoes in developing value-added food products such as sponge cakes (Ramya & Anitha, 2020), cupcakes (Weshah & Al-Hafud, 2023), biscuits (Puscas et al., 2023), beverage (Ahmed et al., 2023), fruit candy (Sehrawat et al., 2023), caramel candy (Mahmoud & Azab, 2025), fruit dessert (de Almeida Costa et al., 2020), ice cream (Peres et al., 2024) and fruit leather (Jahan et al., 2022). Fruit leathers are fruit flavored, flexible sheet like snacks product, enriched with nutrients i.e., fiber, carbohydrate, protein (Sarkar et al., 2020), essential minerals like potassium, magnesium, calcium, iron and bioactive compounds e.g. mangiferin, gallic acid, quercetin, catechin, kaempferol (Mphaphuli et al., 2020).

Shelf life, microbial safety and food value-addition are critical areas of focus for researchers and, consumers owing to the increasing demand for natural, nutrient rich, and convenient foods. Limited data are available on the effects of citric acid and sugar in optimizing the shelf life, quality, and microbial and sensory stability of mango fruit leathers and hence, present study was conducted to explore the addition of citric acid, sugar in alone and in and synergy to evaluate its possible effect in mango fruit leathers.

## 2 Material and methods

### 2.1 Procurement of raw materials, reagents and chemicals

Fresh mangoes (10 Kg) were purchased from the local fruits and vegetables market of Multan, Punjab, Pakistan. Sugar was purchased from the local supermarket, Multan, Punjab, Pakistan. All the reagents and chemicals including DPPH (2,2-diphenyl-1-picryl-hydroxyl), Folin–Ciocalteu phenol reagent (FCR), gallic acid, sodium acetate buffer, iodine, sodium hydroxide and citric acid were of analytical grade and obtained from the Merck (Darmstadt, Germany) and Sigma Chemical Co., Ltd. (St. Louise, MO) via a local supplier (i.e., G.M Scientific Store, Multan, Punjab, Pakistan).

### 2.2 Preparation of mango fruit leathers

Freshly purchased, sorted, graded and cleaned mangoes were utilized and cut into smaller chunks and the puree was prepared using a blender (Model No. MJ-W176P, Matsushita Electric Industrial Co., Ltd., Osaka, Japan). Then, puree was blanched at 85 °C for 5 min. Eventually, puree was spread on the tray and dehydrated at 60 °C for 6 h in cabinet dryer (ABM Carbolite, Model No. 4EKF63A-2, Greiffenberger, Germany) as outlined by (Nurhadi et al., 2023). Mango fruit leathers were prepared by the addition of citric acid and sugar i.e., MFL_0_: zero citric acid and sugar, MFL_C_: 0.2 % citric acid, MFL_S_: 10 % sugar, MFL_CS_: 0.2 % citric acid +10 % sugar, respectively. Thereafter, mango leathers were rolled in butter paper and then packed in polyethylene zipper bags and stored at room temperature for further investigation.

### 2.3 Nutritional and textural composition determination of mango fruit leathers

Chemical composition of mango fruit leather consisting of moisture, crude protein, ash, and crude fiber content were investigated by following established procedures i.e., 934–01, 984–13, 942–05, and 978–10, respectively as outlined by Association of Official Analytical Chemists (AOAC). Moreover, the estimation of minerals i.e., potassium and magnesium were investigated as outlined by (Waseem et al., 2022). The vitamin C, reducing sugar and total soluble solids were determined by the procedures described by (Khan et al., 2023). Moreover, texture of fruit leather including hardness and cohesiveness were investigated by using the texture analyzer (Model: TA. XT Express, Stable Micro Systems, Surrey, UK). The system was standardized by choosing “calibrating force” and then clicked on “probe selection” upon computer and attached 5 mm flat probe with the equipment. Afterwards, sample was positioned on the aluminum base in the center of probe and clicked on “run test” on computer. The probe moved with the speed of 2.5 mm/s and allowed to compress the leather and results were recorded (Sarkar et al., 2021).

### 2.4 2,2-diphenyl-1-picrylhydrazyl (DPPH), total flavonoids content (TFC) and total phenolic content (TPC) determination of mango fruit leathers

#### 2.4.1 Sample extract preparation

For the antioxidant analyses, precisely weighed 1.0 g of each mango fruit leather sample was mixed with 10 mL of distilled water and homogenized thoroughly. The mixture was vortexed and then allowed to stand in a shaking water bath at room temperature for 30 min to facilitate the extraction of antioxidant compounds. After extraction, the samples were centrifuged at 4000 rpm for 10 min, and the supernatant was collected. The extract was further filtered through Whatman No. 1 filter paper, and the clear filtrate was used for all subsequent antioxidant assays, followed by the procedure with slight modifications (Baliyan et al., 2022).

#### 2.4.2 2,2-diphenyl-1-picrylhydrazyl (DPPH)

The DPPH radical scavenging activity of mango fruit leather extracts was determined as outlined by Alam et al. (2025) with slight modifications. A freshly prepared 0.1 mM DPPH solution in methanol was used. For analysis, 2 mL of DPPH solution was mixed with small amount of the sample extract (i.e., 1 mL) in a test tube. The mixture was vortexed and incubated in the dark for 1 h at 27 °C. A control solution consisting of DPPH solution and methanol was prepared under identical conditions. The absorbance of sample and control was recorded at 517 nm using a UV–Vis double-beam spectrophotometer (Specord 200 Plus, Analytikjena, Germany) (Kalsi et al., 2025).

#### 2.4.3 TFC determination

TFC of each sample of mango leather was investigated according to the procedure as outlined by Aleksieva et al. (2025). Accurately measured 0.5 mL of each sample was mixed in 2 mL of distilled water and 0.15 mL of 5 % sodium nitrite solution and then placed in incubator for 6 min. Afterwards, 0.15 mL of 10 % aluminum chloride solution was introduced into the mixture and again incubated for 6 min. Following this, 4 % sodium hydroxide solution was added to the mixture. Volume of the mixture was diluted up to 5 mL by introducing methanol while mixing and then incubated for 15 min. The absorbance was recorded at 510 nm by using spectrophotometer (Perkin-Elmer λ15 UV–vis spectrophotometer, Norwalk, CT) and results were stated as catechin equivalent CE mg/g.

#### 2.4.4 TPC determination

The amount of TPC of all mango leather samples were investigated according to the Folin-ciocalteu method as performed by Ali et al. (2025). Precisely quantified 1.0 g sample was extracted with 10 mL of distilled water. 200 μL sample were dispensed into test tubes containing 1.0 mL of Folin-Ciocalteu's reagent and 0.8 mL of 7.5 % sodium carbonate. The tubes were stirred and set aside for 30 min. The absorption was recorded at 765 nm via spectrophotometer (Perkin-Elmer λ15 UV–vis spectrophotometer, Norwalk, CT). The TPC results were stated as gallic acid equivalents (GAE) in mg/g.

### 2.5 SEM estimation of mango fruit leathers

To evaluate the surface morphology of mango fruit leather, SEM analysis was carried out by employing the scanning electron microscope (SEM Cube II, Emcraft South Korea) conducted at Nano-Materials and Bio-Sensing Research Center (NBRC), Faisalabad. Fresh samples were encased with 3 nm layer of Au—Pd to amplified the conductivity prior to imaging. Afterwards, samples were then examined under an electron microscope at accelerating voltage and emission current i.e., 5.00 kV and 12 μA, with miscellaneous magnifications (Neog et al., 2023).

### 2.6 Measurement of chromatic profile of mango fruit leathers stored at 0–90 days of storage

The surface color of mango fruit leathers was analyzed by using a hand held chromameter (CR-410, Konica Minolta, Osaka, Japan) after standardization with black and white plates. The values were reported in terms of lightness (*L*^*⁎*^), redness (*a*^*⁎*^) and yellowness (*b*^*⁎*^) as outlined by (Li et al., 2025).

### 2.7 pH determination of mango fruit leathers stored at 0–90 days of storage

Precisely measured 1.0 g of fruit leather sample was mixed with 10 mL of distilled water to prepare test solution. A digital pH meter (Ohaus corporation, USA, Model: ST3100) was employed to estimate the pH value of samples after standardization with buffer solution of pH 4 and 7. The pH value was estimated directly by incorporating electrode into the test solution as outlined by (Özkan Karabacak, 2025).

### 2.8 Titratable acidity estimation of mango fruit leathers stored at 0–90 days of storage

Precisely measured 1.0 g of mangoes fruit leather were dissolved with 10 mL of distilled water. Then, 2–3 drops of phenolphthalein indicator were introduced into this test solution. Afterwards, the solution was titrated against solution of 0.1 N NaOH as far as, the light pink color appeared. The results were stated by following below formula as outlined by (Saleem et al., 2023).

$$\text{Titratable Acidity}\;\left(\%\right)=\frac{0.1\times \mathrm{Eq}.\text{weight of acid}\times \text{Normality of NaOH}\times \text{Volume of NaoH used for titration}\;}{\text{Volume of sample}}\times 100$$

### 2.9 Total plate counts, mold and yeast counts determination of mango fruit leathers stored 0–90 days of storage

Precisely quantified 1.0 g of each sample was homogenized and thoroughly mixed in 10 mL of distilled water. Then 1 mL of each sample were sequentially diluted up to 10^−3^ by using 9 mL saline water. Afterwards, nutrient agar and sabouraud dextrose agar (SDA) were prepared for the determination of total plate counts and total fungal counts (i.e., mold and yeast) (Raza et al., 2025). The media was introduced into different petri plates and set aside for solidify. Eventually, 1 mL from each sequentially diluted sample were spread on media petri plates using different spreaders. Media plates were incubated for 24 h at 37 °C. Colonies counts were scrutinized by using colony counter (Infitek Co., Ltd. Model CC-J2 Shandong, China) and results were stated in Log_10_ CFU/g (Muzamil et al., 2025).

### 2.10 Sensory evaluation of mango fruit leathers stored at 0–90 days of storage

Sensory evaluation of mango fruit leather samples was performed by employing 9-point hedonic scale i.e., 9 (strongly liked) and 1 (strongly disliked). Thirty semi-trained experts (*n* = 30) participated in the sensory evaluation at Faculty of Food Science and Nutrition (FFSN), Bahauddin Zakariya University (BZU), Multan. Sensory experts assigned scores to different samples of mango fruit leather depend on their preference. Noise-free and safe environment with proper access to ample light and drinking water were acquired during the sensory evaluation period. Further, Sensory evaluation form was used to record sensory evaluation data in terms of color, taste, texture, odor and overall acceptability (Chong et al., 2025).

### 2.11 Statistical analysis

All experiments were conducted in triplicates (n = 3) and the outcomes are reported as mean values ± standard deviation (S·D). All the data were subjected to analysis of variance (ANOVA) using Statistix 8.1 software (Analytical Software Co., St. Paul, MN, Thalassa, USA). To analyze significant differences among the treatments, least significance difference (LSD) method was employed at *p < 0.05* and 5 % level of confidence.

## 3 Result and discussion

### 3.1 Effect of citric acid and sugar addition on the nutritional and textural properties of mango fruit leather

The results for the nutritional composition of the leathers reported significant increase in ash, K, Mg and vitamin C contents from 1.98 to 2.1 g/100 g, 270–275, 20.7–22.6 and 22–24 mg/100 g in MFL_0_-MFL_CS_ (i.e., zero citric acid and sugar – 0.2 % citric acid+10 % sugar), respectively (Table 1). However, sample MFL_C_ (i.e., 0.2 % citric acid) showed best results for ash, K, Mg and vitamin C contents i.e., 2.2 g/100 g, 281.5, 25.2 and 26 mg/100 g when compared to all other samples, respectively. Previous studies have reported comparable findings for value-added fruit leathers. For instance, Yenrina and Putra (2023) reported *Clitoria ternatea* fruit sheet prepared with 0.2–0.8 % citric acid showed significant increment in ash content i.e., 0.21–0.34 g/100 g respectively. Khaliq et al. (2018) reported mango fruit leather prepared with addition of 9 % sucrose delineated K and vitamin C content i.e., 182.04 and 24.75 mg/100 g as compared to control i.e., 182.15 and 30.81 mg/100 g. Kosasih et al. (2023) reported guava jelly prepared with addition of 0–2 % citric acid elucidated significant increase in vitamin C from 7.3 to 7.7 mg/100 g. The increment in ash and minerals content could be associated to the presence of organic and inorganic compounds in citric acid which during heating processing are burnt, leaving behind the inorganic residues (Khan et al., 2023; Yenrina & Putra, 2023). Also, the increase in vitamin C could be associated with the addition of citric acid, produced acidic condition that contribute in increasing the stability of vitamin C (Kosasih et al., 2023). Total soluble solids (TSS) and reducing sugars were found higher in MFL_S_ (10 % sugar) i.e., 74.7 and 15.07 g/100 g, respectively (Table 1). Earlier studies showed similar findings, reported leather prepared with 9 % sugar showed higher brix and reducing sugar i.e., 68.41°Brix and 45.02 g/100 g, than control i.e., 65.5 and 13.5 g/100 g (Khaliq et al., 2018). In another study, lapsi fruit leather prepared with 27–50 % sugar reported increase in TSS i.e., 50–70°Brix as compared to the sample with 20 % sugar i.e., 45°Brix (KC et al., 2022). The increment in values of reducing sugar and TSS could be linked to the addition of sugar, as it contains high soluble solids and contribute in overall sweetness of the product (KC et al., 2022). The textural properties of mango fruit leathers were investigated, wherein, hardness level for MFL_0_, MFL_C_, MFL_S_, MFL_CS_ were found to be 8.6, 8.7, 3.9 and 5.6 N, respectively (Table 1). Among all treatments, MFL_S_ (i.e., 10 % sugar) demonstrated lowest hardness value (i.e., 3.9 N), while, MFL_C_ (i.e., 0.2 % citric acid) showed highest hardness value (i.e., 8.7 N). Moreover, cohesiveness increased significantly i.e., 1.001–1.472 for MFL_C_-MFL_CS_ than control MFL_0_ i.e., 0.731, respectively. Earlier studies, elucidated comparable findings, wherein, Shende et al. (2020) reported mango leather incorporated with 1 % pectin showed higher hardness and cohesiveness i.e., 8.3 N and 0.67 than control i.e., 7.6 N and 0.55. Nurhadi et al. (2023) reported mango leather incorporated with plasticizers (i.e., 3 % glycerol-5 % sorbitol) showed increment in hardness i.e., 37.5–41.8 N against control i.e., 26.9 N. Roskhrua and Kitchaicharoen (2020) reported mulberry leather incorporated with 0.3–1 % citric acid showed significant increase in cohesiveness i.e., 0.87–0.99 than control i.e., 0.85. The increment in hardness value of mango fruit leather could be linked with addition of citric acid, released hydrogen ion and disrupt hydrogen bonding of water molecules, results in lower moisture, ultimately leads to increase in hardness (Yenrina & Putra, 2023). However, the reduction in hardness value is good for its sensory characteristics could be associated with the addition of sugar, which act as a softening agent and interferes with molecules of leather and uphold water results in less rigidity and more flexible texture (Saad et al., 2021; Sukasih & Widayanti, 2022).

**Table 1:** Effect of citric acid and sugar addition on the nutritional and textural properties of mango fruit leather.

Treatments	Moisture (g/100 g)	Protein (g/100 g)	Ash (g/100 g)	Fiber (g/100 g)	TSS (°Brix)	Reducing Sugar(g/100 g)	Potassium(mg/100 g)	Magnesium(mg/100 g)	Vitamin C(mg/100 g)	Hardness (N)	Cohesiveness
MFL_0_	17.1 ± 0.0^a^	2.2 ± 0.0^a^	2.0 ± 0.0^c^	2.2 ± 0.0^a^	65.9 ± 0.2^c^	11.1 ± 0.0^c^	270.1 ± 0.0^c^	20.7 ± 0.0^c^	22.0 ± 0.0^c^	8.6 ± 0.1^a^	0.7 ± 0.0^d^
MFL_C_	15.6 ± 0.0^c^	2.1 ± 0.0^b^	2.2 ± 0.0^a^	2.0 ± 0.0^b^	61.6 ± 0.0^d^	10.0 ± 0.0^d^	281.5 ± 0.0^a^	25.2 ± 0.1^a^	26.0 ± 0.0^a^	8.8 ± 0.1^a^	1.0 ± 0.0^c^
MFL_S_	16.0 ± 0.0^b^	2.1 ± 0.0^c^	1.9 ± 0.0^d^	1.9 ± 0.1^c^	74.7 ± 0.0^a^	15.0 ± 0.0^a^	268.0 ± 0.0^d^	19.4 ± 0.0^d^	20.3 ± 0.0^d^	3.9 ± 0.1^c^	1.2 ± 0.0^b^
MFL_CS_	15.0 ± 0.0^d^	2.0 ± 0.0^d^	2.1 ± 0.0^b^	1.8 ± 0.0^c^	72.0 ± 0.0^b^	13.1 ± 0.0^b^	275.1 ± 0.0^b^	22.6 ± 0.0^b^	24.0 ± 0.0^b^	5.6 ± 0.1^b^	1.5 ± 0.1^a^

Values are expressed as means (*n* = 3) ± standard deviation (S·D). Small letters exhibiting significant results among means in a column at *p < 0.05*. MFL_0_ = Mango fruit leather with zero citric acid and sugar, MFL_C_ = MFL with 0.2 % citric acid, MFL_S_ = MFL with 10 % sugar, MFL_CS_ = MFL with 0.2 % citric acid and 10 % sugar.

### 3.2 Effect of citric acid and sugar addition on the total phenolic content, flavonoids content and DPPH scavenging activity of mango fruit leather

**Fig. 1:**
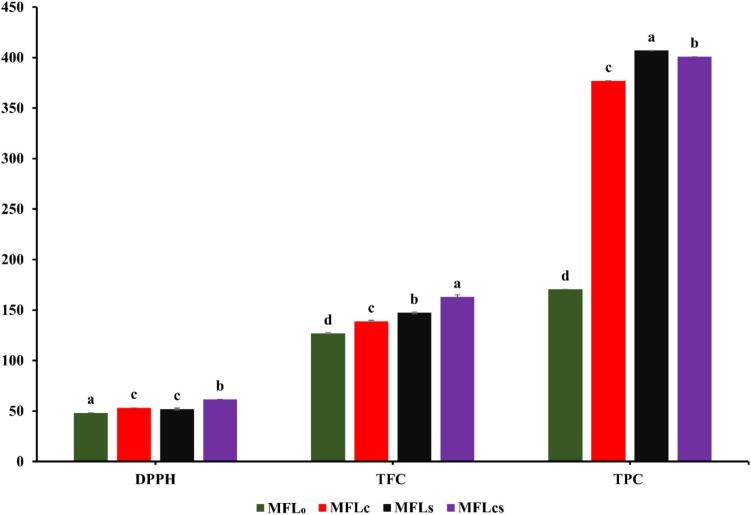
Effect of citric acid and sugar addition on the total phenolic content, flavonoids content and DPPH scavenging activity of mango fruit leather. Results are demonstrated as average value ± standard deviation (SD). DPPH = 2,2-diphenyl-1-picrylhydrazyl, TPC = Total phenolic content, TFC = Total flavonoids content. MFL_0_ = Mango fruit leather with zero citric acid and sugar, MFL_C_ = MFL with 0.2 % citric acid, MFL_S_ = MFL with 10 % sugar, MFL_CS_ = MFL with 0.2 % citric acid and 10 % sugar.

Antioxidant activities of mango fruit leathers showed significant increase (*p < 0.05*) in values of total flavonoids content, total phenolic content and DPPH scavenging activity for MFL_C_ - MFL_CS_ (i.e., 0.2 % citric acid – 0.2 % citric acid+10 % sugar) from 138.8 to 163 mg CE/g, 377–401 mg GAE/g, and 53.2–61.5 %, respectively (Fig. 1). However, the control MFL_0_ (i.e., zero citric acid and sugar) showed significant lower values of total flavonoids content, total phenolic content and DPPH scavenging activity i.e., 126.7 mg CE/g, 170.4 mg GAE/g, and 48.2 % respectively. The sample with 0.2 % citric acid and 10 % sugars elucidated highest total flavonoids (i.e., 163 mg CE/g), total phenolic content (i.e., 401 mg GAE/g) and DPPH activity (i.e., 61.5 %), while the control sample showed lowest flavonoids (i.e., 126.7 mg CE/g), phenolic content (i.e., 170.4 mg GAE/g) and DPPH activity (i.e., 48.2 %), among all samples. The results reported that the addition of citric acid and sugar in mango fruit leather samples showed significant increase (*p < 0.05*) in total flavonoids, total phenolic content and DPPH scavenging activity as compared to the control sample. Earlier, studies available in literature, reported comparable findings for the total flavonoids, total phenolic and DPPH values of fruit leathers, wherein, Mphaphuli et al. (2020) investigated mango fruit leather and reported total phenolic and flavonoids content were found to be 150 mg GAE/g and 49 mg/kg, respectively. Abdrabou (2023) investigated tamarind-carrot based mixed fruit leather prepared with 10 % sugar demonstrated total phenolic content and DPPH scavenging activity i.e., 62.7 mg GAE/g and 91 %, respectively. KC et al. (2022) reported lapsi fruit leather prepared with 20 % sugar showed total flavonoids content and DPPH scavenging activity i.e., 37.6 mg QE/g and 43.2 %, respectively. Ayalew and Emire (2020) reported *Annona muricata* L. fruit leather prepared with *Avena sativa* flour demonstrated total phenolic and flavonoids content i.e., 32.57 mg GAE/g and 4.83 mg CE/g. Jethwani et al. (2020) reported mango fruit bar prepared with 5 % sugar and 0.2 % citric acid showed total phenolic, flavonoids content and DPPH i.e., 71.2 mg GAE/g, 56.7 mg QE/g and 80 %, respectively. The increase in phenolic and flavonoid content may be attributed to the antioxidative properties of citric acid and the protective effect of sugar, which prevents the degradation of these compounds (Castilla-Ramírez et al., 2025; Kushargina et al., 2024). While, the increase in DPPH scavenging activity could be linked with the presence of higher phenolic compounds in treated samples (Kilel et al., 2019). Variations in results may depend on fruit type and variety, processing temperature and time, and interactions among chemical components (KC et al., 2022; Ohijeagbon et al., 2024).

### 3.3 Effect of citric acid and sugar addition on the scanning electron microscopy (SEM) analysis of mango fruit leather

**Fig. 2:**
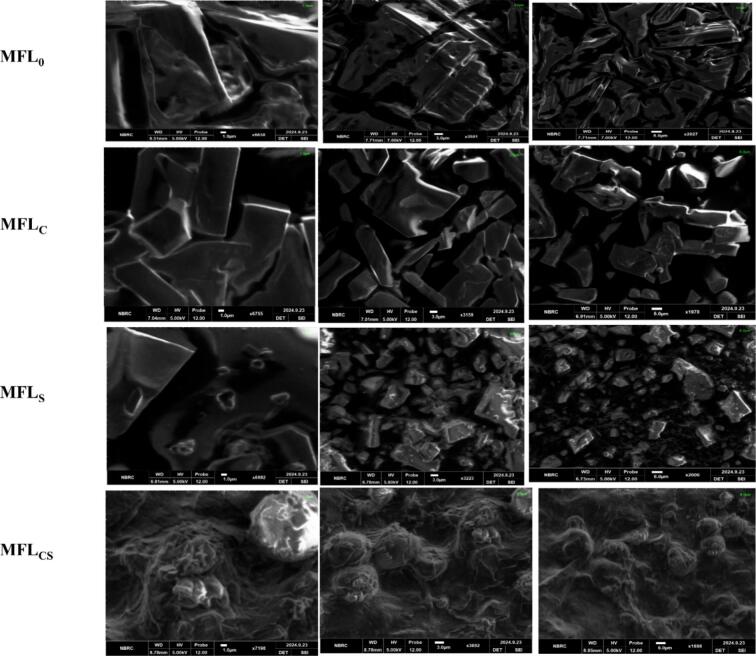
Effect of citric acid and sugar addition on the surface morphology of scanning electron microscopy analysis of mango fruit leather. MFL_0_ = Mango fruit leather with zero citric acid and sugar, MFL_C_ = MFL with 0.2 % citric acid, MFL_S_ = MFL with 10 % sugar, MFL_CS_ = MFL with 0.2 % citric acid and 10 % sugar.

Scanning electron microscope often used for the determination of structure and surface attributes of material and food products (Niro et al., 2022). SEM analysis reported electron microscopic images with different magnifications and resolutions that demonstrated the surface morphology and structural composition of mango fruit leathers prepared with the addition of citric acid and sugar (Fig. 2). The results depicted significant structural and morphological differences among the samples of mango fruit lathers, wherein, sample MFL_0_ elucidated compact, large, irregular shaped, sharp edges and layered structure with minimal porosity. The minimal pores and compact structural surface could be associated with tightly bound matrix due to interaction of hydrogen bond-hydroxyl group i.e., (-OH) and presence of natural fruit sugar as reported by (Nurhadi et al., 2023). Mango fruit leather treatment MFL_C_ (i.e., 0.2 % citric acid) demonstrated well defined crystalline structure due to polysaccharide interaction of citric acid. The slightly roughness appeared on the surface could be due to enhanced phase separation and the leather surface showed significant increase in porosity could associated with disruption of interaction between fibers and sugar due to induction of citric acid as reported by (Melesse & Emire, 2022). Mango fruit leather treatment MFL_S_ (i.e., 10 % sugar) depicted small and dispersed crystalline structure with small number of rough patches. The surface showed reduction in compactness, leading to more fragmented and pores structures. Moreover, sugar granules appeared on the surface morphology and development of small cavities could be linked with the sugar recrystallization and moisture loss because of drying process (Basdemir et al., 2024). Furthermore, the treatment MFL_CS_ (i.e., 0.2 % citric acid+10 % sugar) depicted fluffy, highly porous, fiber like surface morphology. The sample surface exhibited filamentous network and hollow cavities reported significant changes in structural morphology could be associated with the rupturing of cavities containing molecules of water entrapped by sugar and citric acid. Moreover, the spongy and aerated surface of sample could be due to the combination of citric and sugar that results in the disruption of mango matrix as reported in (Das et al., 2021). Some samples of mango fruit leathers exhibited rough structural surface or patches and white spots appeared that could be associated with the existence of fiber that have not been completely homogenized and weak interaction of intermolecular bonds as reported by (Fahrullah et al., 2020; Nurhadi et al., 2023).

### 3.4 Effect of citric acid and sugar addition on the chromatic profile of mango fruit leather stored at 0–90 days

The chromatic profile of mango fruit leathers prepared with the addition of citric acid and sugar showed significant (*p < 0.05*) reduction in *L*^*⁎*^, *a*^*⁎*^ and *b*^*⁎*^ values from 51.6 to 46, 16.9–12.2 and 31.3–16.3 for MFL_C_-MFL_CS_ (i.e., 0.2 % citric acid – 0.2 % citric acid+10 % sugar) at storage of 0–90 days, respectively (Table 2). However, the *L*^*⁎*^, *a*^*⁎*^ and *b*^*⁎*^ values elucidated significant reduction from 52.8 to 41.3, 16.9–12 and 29.3–18.3 for control MFL_0_ (i.e., zero citric acid and sugar) at 0–90 days of storage, respectively. This research delineated best results for the colors of mango fruit leathers in MFL_C_ (i.e., 0.2 % citric acid) for *L*^*⁎*^, *a*^*⁎*^ and *b*^*⁎*^ values from 51.6 to 46, 16.9–13.3 and 31.3–20.8, while the sample developed with zero citric acid and sugar (i.e., MFL_0_) elucidated lowest the *L*^*⁎*^, *a*^*⁎*^ and *b*^*⁎*^ values from 52.7 to 41.3, 16.9–12 and 29.3–18.3, when stored at 0–90 days of storage, respectively. The results clearly indicated that the addition of citric acid and sugar increased the chromatic profile i.e., *L*^*⁎*^, *a*^*⁎*^ and *b*^*⁎*^ values of fruit leather developed with the utilization of mangoes, as compared to the sample prepared with zero citric acid and sugar. Earlier studies available in literature demonstrated comparable findings for the color values of fruit leathers on different storage days, wherein, Khaliq et al. (2018) reported mango fruit leather incorporated with 0–9 % sucrose and skim milk powder showed significant reduction in *L*^*⁎*^, *a*^*⁎*^ and *b*^*⁎*^ values from 47.8 to 37.4, 8.8–8.1 and 38.5–33.1, when stored at 0–75 days, respectively. da Silva Simão et al. (2022) reported strawberry fruit leather showed significant decrease in *L*^*⁎*^, *a*^*⁎*^ and *b*^*⁎*^ from 56.4 to 54, 37.8–30.9, and 21.9–20 at 0–90 days of storage, respectively. Giacalone et al. (2019) reported *Actinidia arguta* fruit leather elucidated significant reduction in *L*^*⁎*^ and *b*^*⁎*^ values from 54.1 to 40 and 6.3–2.5 at storage of 0–21 days, respectively. Buvé et al. (2018) reported significant reduction in *a*^*⁎*^ values from 37.5 to 15 for a fruit-based products at storage of 224 days, respectively. The reduction in *L*^*⁎*^, *a*^*⁎*^ and *b*^*⁎*^ values of mango fruit leathers could be associated with the oxidation, enzymatic browning and pigment degradation due to exposure of light, oxygen and temperature changes over the storage period (da Silva Simão et al., 2022; Khaliq et al., 2018).

**Table 2:** Effect of citric acid and sugar addition on the chromatic profile (i.e., *L*^*⁎*^, *a*^*⁎*^, *b*^*⁎*^) of mango fruit leather stored at 0–90 days of storage.

Days				
	0th	30th	60th	90th
L* values				
MFL0	53 ± 1^a^	47 ± 0^d^	44 ± 2^f-h^	41 ± 1^gh^
MFLC	52 ± 1^ab^	47 ± 3^de^	46 ± 2^d-f^	46 ± 1^d-f^
MFLS	50 ± 1^bc^	47 ± 1^cd^	41 ± 2^h^	44 ± 2^e-g^
MFLCS	49 ± 0^bc^	45 ± 2^d-f^	46 ± 2^d-f^	46 ± 0^d-f^
a* values				
MFL0	17 ± 0^a^	14 ± 1^b-d^	14 ± 1^b-d^	12 ± 1^f^
MFLC	17 ± 2^a^	17 ± 1^a^	14 ± 1^bc^	13 ± 1^bc-f^
MFLS	16 ± 1^a^	13 ± 1^c-f^	12 ± 1^ef^	12 ± 0^f^
MFLCS	15 ± 1^b^	14 ± 1^b-e^	13 ± 1^def^	12 ± 1^ef^
b* values				
MFL0	29 ± 2^ab^	25 ± 1^c^	20 ± 1^d-f^	18 ± 0^e-g^
MFLC	31 ± 2^a^	26 ± 2^bc^	22 ± 1^cd^	21 ± 1^de^
MFLS	26 ± 2^bc^	21 ± 2^de^	19 ± 2^d-g^	17 ± 1^fg^
MFLCS	25 ± 2^c^	20 ± 2^d-f^	18 ± 0^e-g^	16 ± 1^g^

Values are expressed as means (n = 3) ± standard deviation (S·D). Small letters exhibiting significant results among mean values on storage in rows at *p < 0.05*. MFL_0_ = Mango fruit leather with zero citric acid and sugar, MFL_C_ = MFL with 0.2 % citric acid, MFL_S_ = MFL with 10 % sugar, MFL_CS_ = MFL with 0.2 % citric acid and 10 % sugar.

### 3.5 Effect of citric acid and sugar addition on the pH and titratable acidity of mango fruit leather stored at 0–90 days

**Fig. 3:**
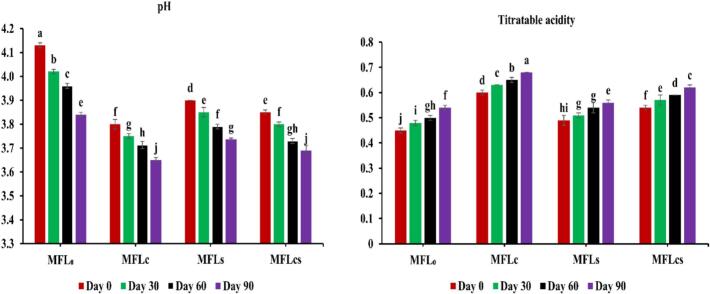
Effect of citric acid and sugar addition on the pH and titratable acidity of mango fruit leather stored at 0–90 days of storage. MFL_0_ = Mango fruit leather with zero citric acid and sugar, MFL_C_ = MFL with 0.2 % citric acid, MFL_S_ = MFL with 10 % sugar, MFL_CS_ = MFL with 0.2 % citric acid and 10 % sugar.

The pH and titratable acidity results of mango fruit leathers prepared with the addition of citric acid and sugar, demonstrated significant (*p < 0.05*) decrease in pH values from 3.80 to 3.69 and increase in titratable acidity values from 0.60 to 0.62 for MFL_C_-MFL_CS_ (i.e., 0.2 % citric acid – 0.2 % citric acid+10 % sugar) at storage of 0–90 days, respectively (Fig. 3). However, the control sample (i.e., zero citric acid and sugar) showed significant (*p < 0.05*) reduction in pH values i.e., 4.13–3.84 and increment in acidity values i.e., 0.45–0.54 for MFL_C_-MFL_CS_ at storage days of 0–90, respectively. The sample prepared with the addition of 0.2 % citric acid delineated lowest pH (i.e., 3.65) and highest titratable acidity (i.e., 0.68), while the sample prepared with zero citric acid and sugar exhibited highest pH (i.e., 3.84) and lowest titratable acidity (i.e., 0.54), as compared to other samples, over the prolonged storage of 90th days, respectively. Though the pH was decreased over the prolonged storage of 0–90 days, however, the fruit leather samples prepared with the addition of citric acid and sugar showed lower pH values as compared to the control sample. As contrast to this, the titratable acidity delineated increment over the prolonged storage of 0–90 days, and the samples developed with the addition of citric acid and sugar exhibited higher titratable acidity values as compared to control, respectively. Earlier studies available in literature reported, showed comparable results for pH and titratable acidity of fruit leathers, wherein, Atif and Mishra (2019) reported mixed fruit leather incorporated with 200 g sugar elucidated significant reduction in pH values from 4.8 to 4.23 and increment in titratable acidity from 0.8 to 0.96, when stored at 0–100 days of storage, respectively. Similarly, in another study, Chiku-guava mixed fruit leather were prepared with 10 % sugar and 2 % pectin reported significant decrease in pH values from 3.8 to 3.6 and increment in titratable acidity from 1.2 to 1.4 at storage of 0–90 days, respectively (Khan et al., 2023). The reduction of pH values over the prolonged storage could be associated with the degradation of organic acids due to greater exposure of oxidation, light, heat or microbial contamination and enzymatic reactions, which may leads to increment of acidity in samples (Habiba et al., 2021; Matenanga, 2020).

### 3.6 Effect of citric acid and sugar addition on the total plate count, mold and yeast count of mango fruit leather stored at 0–90 days

The total plate count results of mango fruit leathers prepared with the addition of citric acid and sugar showed significant (*p < 0.05*) reduction in increase of counts from 1.9 to 3 Log_10_ CFU/mL for MFL_C_-MFL_CS_ (i.e., 0.2 % citric acid – 0.2 % citric acid+10 % sugar) at storage days of 0–90, respectively (Table 3). However, control MFL_0_ (i.e., zero citric acid and sugar) delineated significant higher increase (*p < 0.05*) in counts from 2.2 to 3.4 Log_10_ CFU/mL at storage days of 0–90, respectively. Among treated mango fruit leather samples, MFL_CS_ (i.e., 0.2 % citric acid+10 % sugar) demonstrated best results i.e., 1.3–3 at 0–90 days of storage, respectively. Previous studies available in literature reported comparable results for fruit leathers on storage, wherein, Abdrabou (2023) reported carrot-tamarind based mixed fruit leather incorporated with 15 % sugar delineated significant increment in total plate counts from 1.7 to 2.6 at storage days of 0–120, respectively. Singh et al. (2020) reported mixed fruits leather incorporated with 0.06 % potassium metabisulphite demonstrated significant increase in plate counts from 0 to 2 at storage days of 0–120, respectively. Huchchannanavar and Nidoni (2023) reported mixed fruit leather delineated significant increment in total plate counts from 0 to 3 at storage days of 0–60, respectively. Ahmed Bhatti (2020) reported bael-apricot based fruit leather demonstrated significant increase in counts 0–2.3 at storage days of 0–90 days, respectively. Ulusal Bayram (2018) reported grape fruit leather showed significant increase in plate counts i.e., 3–5.95 at storage days of 0–90, respectively. The increment in plate counts over the storage days of 0–90 in mango fruit leathers could depend on the moisture content, storage conditions (i.e., humidity, temperature), contamination during processing and improper handling (Ofoedu et al., 2020). Moreover, the mold and yeast counts demonstrated significant increment (*p < 0.05*) in counts from 0 to 1.7 for MFL_C_-MFL_CS_ at storage days of 0–90, respectively (Table 3). However, the control sample demonstrated significant higher increase (*p < 0.05*) in mold and yeast counts i.e., 0–2 at storage days of 0–90, respectively. The mango fruit leather sample MFL_CS_ (i.e., 0.2 % citric acid+10 % sugar) reported best results i.e., 0–1.7 at 0–90 days of storage as compared to other samples, respectively. Previous research studies in literature demonstrated comparable results of fruit leathers on storage, wherein, Singh et al. (2020) reported mixed fruit leather prepared with 0.06 % significant increase in mold and yeast counts i.e., 0–1.6 at storage days of 0–120, respectively. Singh and Sonkar (2023) reported pineapple fruit leather prepared with 8 % green tea and sugar illustrated significant increase in mold and yeast counts from 1.7 to 5.5 at storage days of 0–45, respectively. Abdrabou (2023) reported mixed fruit leather prepared with 15 % sugar demonstrated no detectable growth of mold and yeast at storage days of 0–60, respectively. Mohamed et al. (2024) illustrated Egyptian mulberry leather showed significant increment in counts of mold and yeast from 0 to 2.1 at 0–180 days of storage, respectively. The reduction in increment of microbial counts could be due to drying process and combined effects with the addition of sugar and acid, that results in preventing the microbial growth (Singh & Sonkar, 2023). However, The increment in counts over the storage days of 0–90 in mango fruit leathers could depend on the moisture content, storage conditions (i.e., humidity, temperature fluctuations), improper handling and improper packaging (Ofoedu et al., 2020).

**Table 3:** Effect of citric acid and sugar addition on total plate counts, mold and yeast counts (Log_10_ CFU/mL) of mango fruit leather stored at 0–90 days of storage.

		Days		
	0th	30th	60th	90th
Total plate counts				
MFL_0_	2.16 ± 0.02^j^	2.97 ± 0.03^e-h^	3.18 ± 0.03^b-d^	3.36 ± 0.01^a^
MFL_C_	1.90 ± 0.05^k^	2.82 ± 0.04^h^	3.01 ± 0.01^e-g^	3.22 ± 0.05^a-c^
MFL_S_	2.0.01 ± 0.02^jk^	2.87 ± 0.03^f-h^	3.08 ± 0.02^c-e^	3.26 ± 0.01^ab^
MFL_CS_	1.33 ± 0.35^l^	2.63 ± 0.10^i^	2.87 ± 0.01^gh^	3.02 ± 0.01^d-f^
Mold and Yeast counts				
MFL_0_	Nil	1.43 ± 0.10^ef^	1.80 ± 0.04^bc^	2.06 ± 0.02^a^
MFL_C_	Nil	1.10 ± 0.17^gh^	1.52 ± 0.07^de^	1.82 ± 0.04^b^
MFL_S_	Nil	1.26 ± 0.24^fg^	1.63 ± 0.06^cd^	1.86 ± 0.03^ab^
MFL_CS_	Nil	1.00 ± 0.00^h^	1.36 ± 0.10^ef^	1.65 ± 0.05^cd^

Values are expressed as means (n = 3) ± standard deviation (S·D). Small letters exhibiting significant results among mean values on storage in rows at *p < 0.05*. MFL_0_ = Mango fruit leather with zero citric acid and sugar, MFL_C_ = MFL with 0.2 % citric acid, MFL_S_ = MFL with 10 % sugar, MFL_CS_ = MFL with 0.2 % citric acid and 10 % sugar.

### 3.7 Effect of citric acid and sugar addition on the organoleptic properties of mango fruit leather stored at 0–90 days

**Fig. 4:**
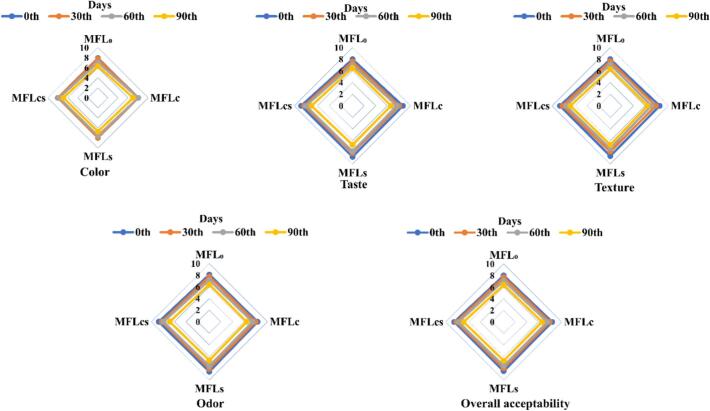
Effect of citric acid and sugar addition on the sensory attributes of mango fruit leather stored at 0–90 days of storage. MFL_0_ = Mango fruit leather with zero citric acid and sugar, MFL_C_ = MFL with 0.2 % citric acid, MFL_S_ = MFL with 10 % sugar, MFL_CS_ = MFL with 0.2 % citric acid and 10 % sugar.

The sensory attributes of mango fruit leathers developed with the addition of citric acid and sugar showed significant (*p < 0.05*) reduction in sensory scores for color, taste, texture, odor and overall acceptability from 8 to 6.9, 8.7–7, 8.5–6.9, 8.3–6.8 and 8.4–6.9 for MFL_C_-MFL_CS_ (i.e., 0.2 % citric acid – 0.2 % citric acid+10 % sugar) at storage days of 0–90, respectively (Fig. 4). However, the control (i.e., zero citric acid and sugar) showed significant lower sensory scores color, taste, texture, odor and overall acceptability from 7.9 to 6.3, 8–6.5, 8–6.3, 8.1–6.4 and 8–6.4 at storage days 0f 0–90, respectively. The sample MFL_CS_ (i.e., 0.2 % citric acid+10 % sugar) illustrated significant (p < 0.05) best sensory scores for color, taste, texture, odor and overall acceptability from 8 to 7, 8.8–7, 8.7–6.9, 8.7–6.8 and 8.5–6.9 at storage days of 0–90, respectively as compared to other treated samples. Various studies available in literature illustrated comparable findings for fruit leathers on storage, wherein, Azeem et al. (2021) reported mango fruit leather prepared with 25 % sugar and 0.06 % citric acid illustrated significant reduction in sensory scores for color, texture, taste, odor and overall acceptability from 6.2 to 5.3, 6.1–5.9, 6.9–5.3, 6–5.3 and 6.85–5 at storage days of 0–90, respectively. Sarkar et al. (2020) reported mango fruit leather illustrated significant reduction in sensory scores for overall acceptability from 8 to 6 at storage days of 0 to 180, respectively. Similarly, in another study Khaliq et al. (2018) reported mango fruit leather prepared with 9 % sugar and skim milk powder delineated significant reduction in sensory scores for color, flavor, texture, taste and overall acceptability from 6.5 to 5.8, 5.8–5.7, 6.2–6, 5.3–5.3 and 6.3–6 at storage days of 0–75, respectively. Similarly, in another study mixed fruit leather demonstrated significant reduction in sensory scores for color, texture, taste and overall acceptability from 7.1 to 7, 7.3–7.2, 7–6.9 and 7.1–7 at storage days of 0–90, respectively (Ahmed Bhatti, 2020). The reduction in sensory scores for color, taste, texture and overall acceptability of mango fruit leather samples over the prolonged storage of 0–90 days could associated with oxidation, moisture content, storage conditions (i.e., temperature and humidity), improper packaging (Ofoedu et al., 2020). The sample containing sugar and citric acid delineated best sensory scores could be linked with the anti-oxidative, color retention property of citric acid and increasing sweetness and softening property of sugar (Saad et al., 2021; Sukasih & Widayanti, 2022).

## 4 Conclusion

Mango and its value-added products are considered as the rich source of essential nutrients such as protein, fiber, ash, minerals and antioxidants, that contributes to overall health and wellness. The present study demonstrates that the combined use of citric acid and sugar is an effective strategy to enhance the nutritional stability, functional properties, and overall acceptability of mango fruit leather during storage. Incorporation of 0.2 % citric acid and 10 % sugar (MFL_CS_) strengthened the product's structural integrity, promoted oxidative stability, and supported favorable microbial behavior over the 90-day period. These improvements highlight the synergistic role of acidification and sweetening agents in maintaining product quality in fruit-based dehydrated snacks. The study emphasizes the potential of controlled formulation to extend shelf life while preserving consumer appeal. Future research should explore similar optimization across diverse fruit matrices and broader concentration ranges to establish more universal formulation guidelines for commercial-scale production.

## CRediT authorship contribution statement

**Muhammad Muzamil:** Writing – review & editing, Writing – original draft, Methodology, Investigation, Formal analysis, Data curation, Conceptualization. **Raheel Suleman:** Writing – review & editing, Validation, Supervision, Formal analysis, Data curation, Conceptualization. **Muhammad Waseem:** Writing – review & editing, Visualization, Software, Investigation. **Tariq Ismail:** Writing – review & editing, Supervision, Resources. **Muhammad Shoaib:** Writing – review & editing, Visualization, Software, Data curation. **Muhammad Qamar:** Writing – review & editing, Visualization, Methodology. **Muhammad Aftab Zahoor:** Writing – review & editing, Visualization. **Tawfiq Alsulami:** Writing – review & editing, Visualization, Validation, Software. **Robert Mugabi:** Writing – review & editing, Visualization, Validation, Software.

## Declaration of competing interest

The authors declare that they have no known competing financial interests or personal relationships that could have appeared to influence the work reported in this paper.

## Data Availability

Data will be made available on request.
